# Efficacy of adding mobilization and balance exercises to a home-based exercise program in patients with ankle disability: a randomized controlled trial

**DOI:** 10.3389/fmed.2025.1512587

**Published:** 2025-02-19

**Authors:** Hadaya Mosaad Eladl, Dalia Mahmoud Abdelmonem Elsherbini, Radwa T. Elshorbagy, Ateya Megahed Ibrahim, Mohamed El-Sherbiny, Sherief El-Sayed Abd El-Farrag Ibrahim, Ghada Ibrahim Fahmi Elfayoumi, Moutasem Salih Aboonq, Yasser M. Elbastawisy, Mohamed El-Dosoky Mohamed Salama, Nesma M. Allam

**Affiliations:** ^1^Department of Physical Therapy and Health Rehabilitation, College of Applied Medical Sciences, Jouf University, Sakaka, Saudi Arabia; ^2^Department of Physical Therapy for Surgery, Faculty of Physical Therapy, Cairo University, Giza, Egypt; ^3^Department of Clinical Laboratory Sciences, College of Applied Medical Sciences, Jouf University, Sakaka, Saudi Arabia; ^4^Department of Physical Therapy for Musculoskeletal Disorders and Their Surgeries, Faculty of Physical Therapy, Cairo University, Giza, Egypt; ^5^College of Nursing, Prince Sattam bin Abdulaziz University, Al-Kharj, Saudi Arabia; ^6^Department of Family and Community Health Nursing, Faculty of Nursing, Port Said University, Port Said, Egypt; ^7^Department of Basic Medical Sciences, College of Medicine, AlMaarefa University, Diriyah, Saudi Arabia; ^8^Department of Rheumatology& Rehabilitation, Mansoura Faculty of Medicine, Mansoura, Egypt; ^9^Department of Basic Medical Sciences, College of Medicine, Taibah University, Medina, Saudi Arabia; ^10^Department of Basic Medical Sciences, College of Medicine, Al-Rayan Colleges, Al-Madinah, Saudi Arabia; ^11^Department of Anatomy, Faculty of Medicine, Mansoura University, Mansoura, Egypt; ^12^Department of Neuroscience Technology, College of Applied Medical Science in Jubail, Imam Abdulrahman Bin Faisal University, Jubail, Saudi Arabia

**Keywords:** mobilization, balance, physical therapy, ankle disability, road traffic accidents

## Abstract

**Background:**

Ankle joint fractures account for 10.2% of all fractures. It has been hypothesized that mobilizing the ankle joint is a crucial intervention for improving balance and range of motion (ROM).

**Objective:**

The current study explores the impact of incorporating mobilization, balance training, and physical therapy exercises into a home-based program on pain, ROM, health-related quality of life (HRQoL), and peak muscle torque in patients with ankle disability following road traffic accidents (RTAs).

**Methods:**

In this single-blinded, randomized controlled trial, 60 participants with post-RTA ankle disability were randomly assigned to either the experimental group or the control group. The experimental group underwent home-based exercises combined with mobilization, balance training, and physical therapy for 2 months, while the control group followed only a home exercise program. The interventions were then implemented 3 days per week. Pain was evaluated using the Visual Analogue Scale (VAS), ankle ROM was measured using a universal goniometer, HRQoL was evaluated using the Short Form (SF 36) survey, and peak torque was assessed using the Biodex System isokinetic dynamometer.

**Results:**

Significant improvements were observed in the experimental group compared to the control group in pain, ROM, HRQoL, and peak muscle torque (*p* < 0.001). After 8 weeks, the experimental group outcomes for VAS, ROM of ankle dorsiflexion/plantarflexion (DF/PF), peak torque of DF/PF, and HRQoL physical and mental component summaries (PCS and MCS) were 2.55 ± 0.22, 13.02 ± 0.38, 25.06 ± 0.40, 34.12 ± 0.81, 47.46 ± 0.90, 43.15 ± 0.78, and 45.01 ± 0.68, respectively. In contrast, the results of the control group were 5.98 ± 0.31, 6.16 ± 0.28, 14.97 ± 0.35, 26.17 ± 0.90, 41.38 ± 0.94, 33.05 ± 1.10, and 34.52 ± 1.06, respectively.

**Conclusion:**

Incorporating mobilization and balance exercises into a physical therapy program significantly improves pain, ankle ROM, HRQoL, and muscle torque (DF/PF) in patients with ankle disability following RTAs.

**Clinical trial registration:**

ClinicalTrials.gov, identifier NCT06010706.

## 1 Introduction

Road traffic accidents (RTAs) pose a significant health risk, particularly in low- and middle-income regions. Road traffic deaths (RTDs) were recorded as the third most common cause of death (1).

Injuries resulting from RTAs exceed 50 million people worldwide yearly, which is considered to be a leading cause of disability (2). The incidence of fractures to the ankle joint has been estimated to be 168.7 per 100,000 people yearly, accounting for 10.2% of all fractures (3). Middle-aged women and young men are the most affected age groups (4). Lateral or medial malleolus fractures and distal fibula or tibia fractures are known as ankle fractures (5).

Ankle traumas have different causes and are most often the result of an indirect pathogenic mechanism. They may be brought on by vertical, rotational, adductive, and abductive forces exerted on the foot in either supination or pronation (6). Stable fractures with no translation or those with minimal movement and no variation in the bone length, when the tarsal bone is positioned correctly with respect to the specific surface of the tibia, are treated by plaster cast as a conservative treatment (7).

Ankle fractures frequently cause stiffness, chronic pain, and functional deficits. The ankle immobilization after a plaster cast usually lasts approximately 6 weeks. Throughout this period, subjects have limitations in ankle range of motion (ROM), lowered peak torque of plantar flexors, atrophy, and decreased central activation of the calf muscle (8). Due to immobilization, young and old age patients suffer from comparable reductions in muscle size, mass, strength, particular force, power, and endurance (9, 10). However, retraining reversed the loss of muscular strength and completely recovered the pre-disuse levels of the muscular features in young patients (10).

Balance deficits are among the most common factors lowering functional performance after an ankle fracture (11). Furthermore, it has been related to some variables directly or indirectly induced by ankle fractures, like reduced muscle strength or limitation in ankle dorsiflexion (DF) and plantar flexion (PF) ROM. These elements are related to variations in the kinematics of the lower extremity, particularly while performing single-leg stance tasks and dynamic sagittal plane activities like single-leg reaching tasks or gait (12, 13). Therefore, balance impairment linked to these variables may change the gait cycle (5, 13). Gait cycle variations during rehabilitation can increase pain, alter joint weight distribution, and raise the risk of falls (5).

Ankle fractures can also have long-lasting social and psychological effects. Adverse social outcomes include difficulty returning to work and dependence on disability benefits. The negative psychological effects have included depression, exhaustion, sleep difficulties, and anxiety (14). All these factors lead to severe restrictions in daily living activities like climbing stairs, walking, running, and squatting. Absence from work for approximately 3 months is common. Disability after ankle fracture persists over a year after cast removal (15).

Manual therapy, including joint mobilization, has been suggested to relieve pain and enhance the flexibility of joint components by actions at the central nervous system, psychological, and regional levels (16). It enhances concurrent stimulation of the sympathetic nervous system (17). Furthermore, joint mobilization can cause improvement in ankle mobility, enhance gait patterns, require fewer sessions of treatment, and allow for a quicker return to activities compared to the standard rest, ice, compression, and elevation regimen (18, 19).

Balance training, known as the capacity to keep the center of body mass throughout the stability restrictions, has been reported to enhance postural control (20), aid in joint ROM restoration, increase muscle strength, and enhance coordination. Balance and proprioception training are crucial since their restoration has a relevant effect on the clinical results of treatment and the quality of life (21).

Relevant studies have explored the effect of active controlled movement (22), joint mobilization and stretching (8), talocrural joint mobilization (23), and weight-bearing plus mobilization (24), as well as the short-term effect of mobilization (25) on disability after ankle fractures, but to the recent knowledge, there are no previous studies investigating the combined effect of mobilization, balance training, in addition to physical therapy exercise programs on pain, ankle ROM, HRQoL, and muscle torque in patients with ankle disability after RTAs. Therefore, this study aimed to evaluate the additional effect of mobilization, balance, and home-based exercise on pain, ankle ROM DF/PF muscle torque, and HRQoL in patients with ankle disability after RTAs.

## 2 Materials and methods

### 2.1 Trial design

The current study is a prospective, single-blinded, parallel-group, randomized controlled trial. It was conducted per the Declaration of Helsinki after obtaining approval from the Research Ethics Committee, Qurayyat Health Affairs (No: 2023–104). We recruited participants from hospitals in the Aljouf region and conducted the study at their outpatient clinics between August 2023 and June 2024. The study had been prospectively registered at ClinicalTrials.gov (NCT06010706).

Participants were informed of the study’s goal and their right to withdraw at any time. Prior to participation, all participants filled out a formal consent form. This study followed the guidelines of CONSORT.

### 2.2 Participants

Participants were screened for eligibility based on the study’s inclusion criteria: participants aged 18–50 years with a stable fracture of the ankle (unimalleolar, bimalleolar, or trimalleolar) sustained in RTAs, conservatively immobilized in a cast with removal within the previous 7 days, approved by an orthopedic consultant for partial or full weight-bearing as tolerated, experiencing at least 4 out of 10 pain, assessed using the VAS in the affected leg when prone to up to 50% of bodyweight, and demonstrating the ability to cooperate. The exclusion criteria included participants with coexisting pathologies or injuries, such as fractures of the contralateral leg, additional fractures on the ipsilateral leg, neurological injuries, malunion or non-union of the syndesmosis, psychiatric diagnoses, drug abuse, systemic diseases, rheumatic conditions, or contraindications to manual therapy.

### 2.3 Randomization

A total of 60 participants with stable ankle fractures were randomly allocated to either the experimental group or the control group using a computer-generated block randomization program at http://www.randomization.com/. Randomization was in blocks of 4 with a 1:1 allocation ratio to decrease the bias. One author, not engaged in recruiting, data collection, or treatment, was responsible for executing the randomization. Randomization codes were contained in sealed opaque envelopes for confidentiality and were consecutively numbered to guarantee concealed allocation.

### 2.4 Intervention

Following baseline measurements, envelopes were unfolded by the first author, who proceeded with the intervention according to the allocation of the groups. The treating author conducted a comprehensive neuromusculoskeletal assessment focused on the ankle and foot. Second, other potential areas and structures, including the lumbar spine, hip, and knee joints, might have a role in the limitation of function. The examination focused on detecting limited motion, muscle weakness, pain, and limited function. Participants in the study group attended an instructional session on exercise progression during enrollment, tailored to their status and the therapist’s judgment.

Group A (experimental group) received mobilization, balance, weight-bearing, stretching, and strengthening exercises, in addition to home-based exercises as described in Supplementary Table SI. In contrast, participants in Group B (the control group) received home-based exercises only. The exercise program was applied for 8 weeks, 3 times per week for 45 min, supervised by the authors, who were well-trained and educated about the treatment protocol and had an average of 6.5 ± 3.1 years of clinical experience in the Faculty of Physical Therapy outpatient clinic.

#### 2.4.1 Mobilization techniques

The joint mobilization techniques used included talocrural joint distraction, which was used as an initial treatment to relieve pain and improve overall ankle joint mobility, talocrural dorsal (posterior) glide to enhance dorsiflexion, and talocrural ventral (anterior) glide to improve plantar flexion. Mobilization techniques were conducted performed within the joint’s pain-free range of DF and PF.

Each exercise involved 3 to 5 sets of 60 s, with a 60-s rest period between sets, gradually increasing in intensity (8, 18). Mobilization was conducted following the Maitland technique grades, ranging from grades I to IV. The distal fibula and tibia were stabilized during the mobilization procedure, while the talus was mobilized anteriorly and posteriorly (26).

#### 2.4.2 Weight bearing and balance exercise

Weight-bearing progressed from partial weight-bearing on the participant’s leg because of pain to complete weight-bearing. The achieved target was set as the optimum weight for the participant to achieve 10 times without a break. Then, the weight gradually increases until the participant reaches their maximum weight-bearing capacity. Each exercise was performed in 3 sets of 10 repetitions daily (27).

The balance training program included a series of progressive exercises performed on different surfaces and under varying conditions. Participants began with balance training on a hard floor, followed by maintaining balance on a balance board in a standing position on both legs and then on one leg, starting with the non-affected left and progressing to the affected leg.

Other exercises included standing on one leg while throwing a ball (first on the non-affected leg and then the affected), walking on even and uneven ground, and performing multidirectional rolling motion between parallel bars while standing on one leg (starting with the non-affected leg and then the affected one). These activities were performed with both open and closed eyes. Each exercise was repeated three times, with a 10-s rest period between repetitions (28).

#### 2.4.3 Stretching exercise

Different types of stretching were used for ankle PF with the knee, both in flexion and extension. Each participant stood on a 10° inclined board with slight knee flexion. The knee was gradually flexed until pain from the stretch was felt at the gastrocnemius and soleus muscles. Stretching was maintained for 20 s for 15 repetitions. In total, 10 s of rest were allowed between stretching exercises (29, 30).

#### 2.4.4 Strengthening exercise

Exercises started resistance-free on the first day of the study and were then followed by elastic-exercise-Thera-Band resistance for the involved ankle joint only under the continuous supervision of the therapist. The progression provided increased resistive exercise as there was a continuous increase in the number of sets. Thera-Band resistance (31).

#### 2.4.5 Home-based exercise program

Participants in both groups received the instructions for the home-based exercise program. It primarily consisted of exercises targeting muscle flexibility and joint ROM (32).

The exercises were selected based on the protocol established by Moseley et al. (33). The exercise program was tailored to meet the individual needs of each participant and included self-stretching exercises for the calf muscles, strengthening exercises for ankle dorsiflexors and plantar flexors, and ROM exercises (as described in Supplementary Table SII). Participants were given exercise recording sheets and cards so they could record daily exercise repetition. The exercise frequency was three sets of 10 repetitions daily. To follow their compliance with the home program, participants had to complete an exercise log. After treatment, participants were advised to continue exercises for further improvement or gain maintenance.

### 2.5 Outcomes

Outcome measures were obtained by an author who was blinded to the group’s allocation. Participants’ demographic data, such as age, level of education, body mass index (BMI), working status, dominant lower limb, injured limb, fractured bone, and immobilization duration, was recorded before the study. The primary outcome was pain, which the VAS assessed. While the secondary outcome measures were ROM for ankle DF and PF, which were measured by the universal goniometer, HRQoL was evaluated by the Short Form 36 Health Survey (SF-36), and the peak torque of ankle DF and PF was measured by the isokinetic dynamometer. All outcomes were procured at baseline and after 8 weeks of intervention.

#### 2.5.1 Pain assessment

Patients used a vertical line drawn horizontally to indicate the pain level on a 10 cm VAS before and after the end of the study. The left end of the VAS accounted for ‘no pain’ and the right end for ‘most severe intolerable pain’ without intermediate categories or descriptive words. During the assessment session, the patient was instructed to stand and distribute weight equally between both lower limbs, and the researcher used a written explanation to provide a standardized introduction to the VAS. After that, the patient was instructed to use the horizontal scale to draw a vertical line (34). VAS has a high response in change detection, as well as high test–retest reliability (intraclass correlation coefficient (ICC) = 0.71–0.99) (35).

#### 2.5.2 Ankle ROM

A standard universal goniometer with a 360° plastic side and 10-inch movable arms was utilized to measure ankle DF and PF passive ROM with the participants maintaining an extended knee position. Evaluators were asked to stand beside each individual’s affected ankle joint. With the participants seated relaxed with full knee extension (0°) while the feet were out of the treatment table, the investigator passively dorsiflexed and plantarflexed the ankle from a neutral position (90° between the tibial shaft and the foot) till a firm end feel was elicited. The goniometer axis was over the center of the lateral malleolus of the fibula, while the fixed and movable arms were aligned parallel to the fibular shaft and the head of the 5th metatarsal, respectively. The evaluator recorded the angle in entire degrees once the evaluator was confident that the measurement had been completed and no movement of the movable arms of the goniometer or from the knee joint occurred throughout the measurement (36). Five measurements were applied in every position, and the mean was calculated (37). ICC for ankle dorsiflexion is (0.12–0.73), and validity (0.51–0.83). Mean difference in reliability and validity ranged from (−2.0° to 3.0°) and from (−6.6° to 7.5°), respectively (38).

#### 2.5.3 Health-related quality of life (HRQoL)

Health-Related Quality of Life (HRQoL) was assessed using the SF-36, which comprises the Physical Component Summary (PCS) and the Mental Component Summary (MCS). Eight domain scales were established: physical function, bodily pain, role-physical, general health, vitality, role-emotional, social functioning, and mental health. The total score varies from 0 to 100, with 0 denoting the worst while 100 represents the best. The Arabic version of SF-36 was utilized for the participants as it is the main language in Saudi Arabia (39). Its reliability is good (ICC = 0.87) and has good construct validity (40).

#### 2.5.4 Peak torque

The Biodex System isokinetic dynamometer (Biodex Inc., Shirley, NY, USA) was used to determine the peak torque of ankle DF and PF. Concentric-concentric contraction of ankle DF/PF with the participants in a semi-recumbent position, while hips were flexed to 90°–100° and their knees in 20°-30° flexion. The dynamometer’s anatomical axis was parallel to that of the ankle dynamometer. Straps at the ankle and forefoot held the foot in place, and straps at the hips, chest, and knee were provided for proximal stability. Three submaximal repetitions and three maximal contractions were applied to familiarize the patient with the device and procedures. This is followed by 2 min of rest, then three repetitions of maximal concentric contractions without a break for the reciprocal muscles in the same direction. In total, 2 min of rest were permitted between each test trial to avoid fatigue. The greatest peak value was tracked for each test repetition (41). The same procedure was repeated after a 5-min rest for the next randomly ordered group of muscles (42). The test angular velocity used was 60^o^.s^−1^. Slower velocity would detect variations in torque capacity (43, 44). Isokinetic reliability for evaluating muscle strength of ankle DF was ICC of 0.77–0.93, and ankle PF was ICC of 0.78–0.95 (45).

### 2.6 Data analysis

#### 2.6.1 Sample size calculation

G*Power software (version 3.1.9.2; Heinrich-Heine-Universität, Düsseldorf, Germany) was utilized to detect the sample size. The data for the study group and control group were used in determining the effect size (d) (22, 46). The effect size (d) (0.79) was applied at 80% power to detect effect sizes with *α* = 5%. Sample sizes for the *T*-test with the two independent groups were 21 for each group. Assumed a maximum drop-out rate of 20%, which causes a sample size of 26 patients/group and a total of 52 participants required for this study. A total of 74 individuals were assigned to participate in the study, as previous related studies typically reported a sample size of 30–31 participants in each group.

#### 2.6.2 Statistical analysis

GraphPad Prism version 9 was used to analyze collected data that were presented as the mean ± SD and mean ± SEM. For detection of the differences in demographic characteristics among the experimental and control groups, the Student’s *t*-test was used. The outcome variables between the two groups were detected utilizing a two-way repeated-measures ANOVA. Qualitative data were presented as a percentage. We used a chi-square test to compare the proportions. A partial eta square was used to calculate the effect size between groups. The significance criterion of *p* < 0.05 was used. The difference between the outcomes within each group was tested using the paired t-test. Prior to applying the parametric assumption, the homogeneity and normality of the variance were statistically assessed.

## 3 Results

### 3.1 Participants flow

**Figure 1:**
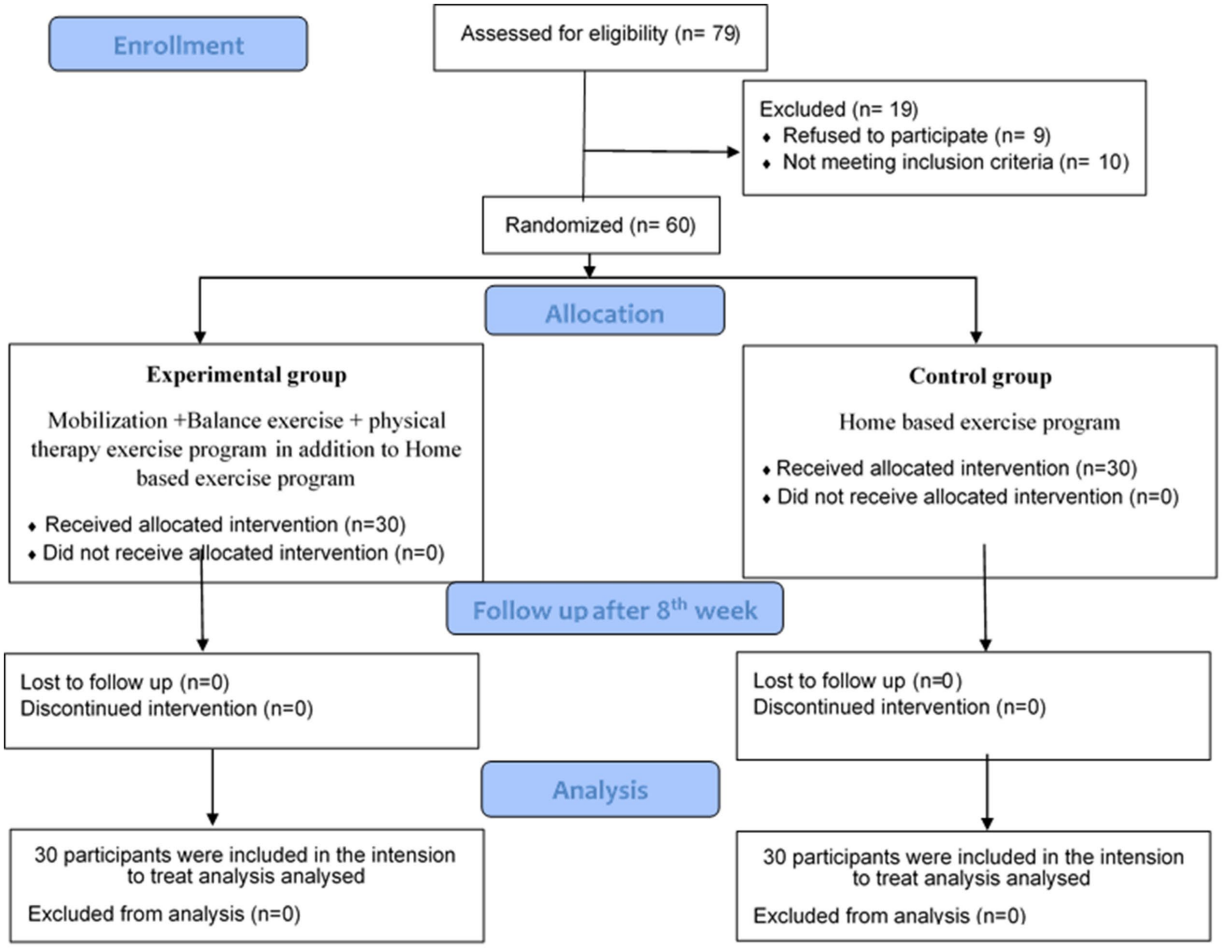
CONSORT flowchart for participant recruitment and allocation.

The flow diagram of the participants is illustrated in Figure 1. The main causes for exclusion were declined participation (*n* = 9) and not meeting the inclusion criteria (*n* = 10).

An intention-to-treat analysis using multiple imputations was conducted to address missing data. One participant reported delayed onset muscle soreness of the gastrocnemius and soleus muscles in the study group, another one complained of temporary pain increase of less than a week, while one participant reported pain in the hamstring muscle after the first session of exercise. Patients were instructed to rest for 2 days and resume treatment after the symptoms resolved.

### 3.2 Baseline data

Table 1 demonstrates the baseline characteristics and patient demographics of the study. Sixty patients were enrolled in the study. Thirty patients constitute the experimental group (mean age 37.55 years; 25 men and women; and mean BMI 26.61 kg/m^2^), and 30 patients constitute the control group (mean age 36.70 years; 27 men and 3 women; and mean BMI 26.52 kg/m^2^) for ankle disability after RTAs.

**Table 1:** Demographic and baseline characteristics of the patients.

Variables	Experimental group (*N* = 30)		Control group (*N* = 30)		*p*-value between groups
	*N* (%) Mean ± SD	*x^2^* (*p*-value)	*N* (%) Mean ± SD	*x^2^* (*p*-value)	
Age (years)	37.55 ± 8.35		36.70 ± 8.01		0.69 (ns)^t^
Gender					
Men	25 (83.3)	13.33 (<0.001^^^)	23 (76.7)	8.54 (<0.01^^)	0.52 (ns)^c^
Women	5 (16.7)		7 (23.3)		
BMI (kg/m^2^)	26.61 ± 2.99		26.52 ± 2.82		
Normal (18.5–24.9)	9 (30)	8.60 (<0.05^)	9 (30)	11.40 (<0.01^^)	0.92 (ns)^c^
Overweight (25–29.9)	17 (56.7)		18 (60)		
Obesity class I (30–34.9)	4 (13.3)		3 (10)		
Level of Education					
Primary	5 (16.7)	3.80 (ns)	4 (13.3)	5.60 (ns)	0.93 (ns)^c^
Secondary	12 (40)		12 (40)		
University	13 (43.3)		14 (46.7)		
Working status					
Working	24 (80)	10.80 (<0.01^^)	25 (83.3)	13.33 (<0.001^^^)	0.72 (ns)^c^
Not working	6 (20)		5 (16.7)		
Dominant lower limb					
Right	26 (86.7)	16.13 (<0.001^^^)	25 (83.3)	13.13 (<0.001^^^)	0.72 (ns)^c^
Left	4 (13.3)		5 (16.7)		
Injured side					
Right	21 (70)	4.80 (<0.05^)	22 (73.3)	6.53	
Left	9 (30)		8 (26.7)	(<0.05^)	0.77 (ns)^c^
Fracture severity*					
Less severe	22 (73.3)	6.53 (<0.05^)	24 (80)	10.80 (<0.01^)	0.54 (ns)^c^
More severe	8 (26.7)		6 (20)		
Duration of immobilization (days)	70.47 ± 9.21		71.90 ± 8.52		0.54 (ns)^t^

BMI, Body mass index; *N*, number.

Quantitative data were presented as mean ± SD, while frequencies and percentages were used to present qualitative data. For categorical variables, a chi-squared test was used to determine the *p*-values, and for continuous variables, an independent student t-test was used.

^*p* value ≤0.05, statistically significant chi-square; (ns)^c^, non-significant chi-square; (ns)^t^: non-significant *t*-test.

*, Less severe = 1 malleoli fractured; more severe = 2 or 3 malleoli fractured or the occurrence of dislocation, irrespective of the number of malleolar fractures.

### 3.3 Clinical measures before and after physical therapy exercise for ankle disability

**Figure 2:**
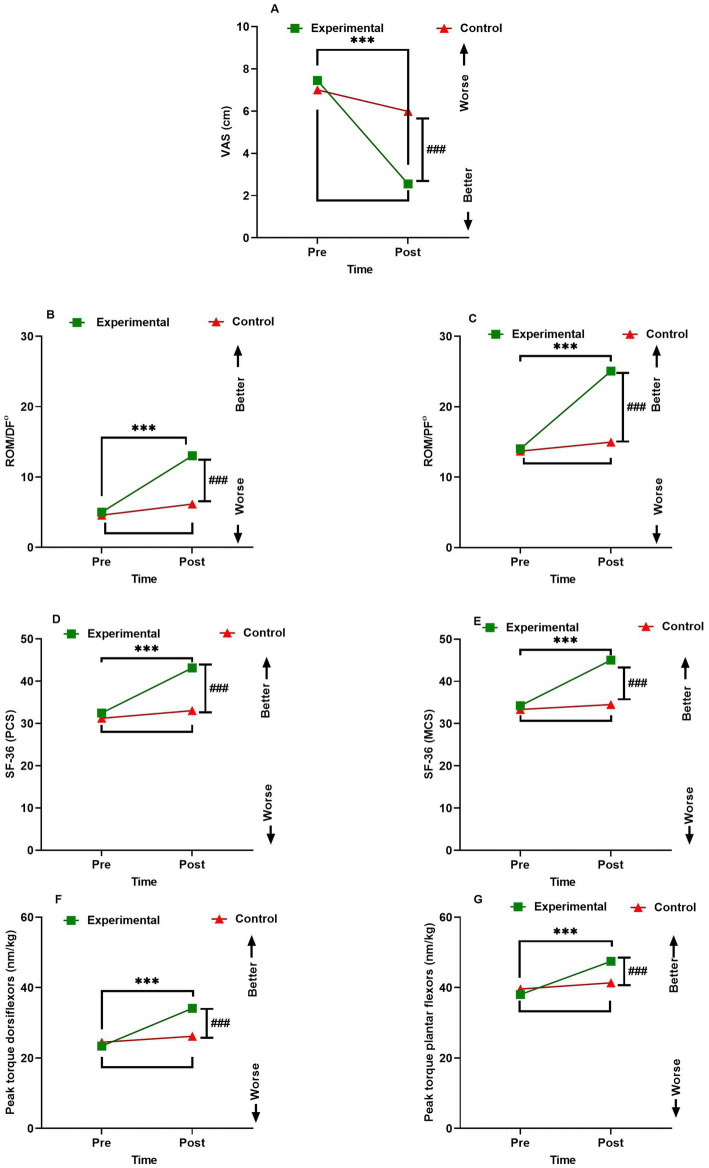
Interactions between group and time for **(A)** VAS (cm) peak torque **(B)** ROM/DF^°^ **(C)** ROM/DF^°^ **(D)** SF-36 (PCS) **(E)** SF-36 (MCS) **(F)** Peak torque dorsiflexors (nm/kg) **(G)** Peak torque plantar flexors (nm/kg). Data are expressed as mean ± SEM. ***p < 0.001 within the experimental group pre *vs.* post-intervention. ^###^ *p* < 0.001 of experimental *vs.* control group post-intervention. ROM/DF^°^, Range of motion dorsiflexion; ROM/DF°, Range of motion plantarflexion; SF-36 (PCS), Short Form 36 (SF-36) Health Survey Physical Component Summary; SF-36 (MCS), Short Form 36 (SF-36) Health Survey Mental Component Summary.

The comparisons of clinical outcomes of the groups are shown in Table 2 and Figure 2. The VAS was statistically significantly lower in favor of the experimental group compared to the control group (*p* < 0.001*001*, *η*^2^ = 0.58). The DF and PF ROM were significantly better post-intervention in the experimental compared to the control group, F(1,58) = 132.55, *p < 0.001*, *η*^2^ = 0.79 for DF, and F(1,58) = 234.45, *p* < 0.001, *η*^2^ = 0.86 for PF. Peak torque DF and PF were significantly improved post-intervention in the experimental compared to the control group, F(1,58) = 25.25, *p* < 0.001, *η*^2^ = 0.43 for DF, and F(1,58) = 20.21, *p* < 0.001, *η*^2^ = 0.27 for PF. SF-36 (PCS) and SF-36 (MCS) were significantly better post-intervention in the experimental compared to the control group, F(1,58) = 14.19, *p* < 0.001, *η*^2^ = 0.49 for SF-36 (PCS), and F(1,58) = 23.55, *p* < 0.001, *η*^2^ = 0.54 for SF-36 (MCS).

**Table 2:** Clinical measures before and after physical therapy exercise for ankle disability after RTAs.

Variable	Experimental group				Control group				Group X Time interaction (F) *p* value	*P*^b)^ between groups	effect size (η^2^)
	Pre	Post	MD (95% CI)	*P*^a)^ within group	Pre	Post	MD (95% CI)	*P*^a)^ within group			
VAS (cm)	7.45 ± 0.28	2.55 ± 0.22	−4.90 (−5.33–−4.4 7)	< 0.001	7.00 ± 0.28	5.98 ± 0.31	−1.02 (−1.22—0.81)	0.65	F_1,58_ = 72.07 < 0.001	< 0.001	0.58
ROM/ DF (degree)	5.01 ± 0.26	13.02 ± 0.38	8.00 (7.49–8.52)	< 0.001	4.58 ± 0.24	6.16 ± 0.28	1.58 (1.38–1.79)	0.45	F_1,58_ = 132.55 < 0.001	< 0.001	0.79
ROM/ PF (degree)	14.02 ± 0.31	25.06 ± 0.40	11.04 (10.41–11.66)	< 0.001	13.71 ± 0.33	14.97 ± 0.35	1.26 (1.04–1.49)	0.52	F_1,58_ = 234.45 < 0.001	< 0.001	0.086
SF-36 (PCS)	32.46 ± 1.09	43.15 ± 0.78	10.69 (9.59–11.79)	< 0.001	31.23 ± 1.13	33.05 ± 1.10	1.82 (1.47–2.17)	0.78	F_1,58_ = 14.19 < 0.001	< 0.001	0.49
SF-36 (MCS)	34.22 ± 1.06	45.01 ± 0.68	10.79 (9.42–12.17)	< 0.001	33.34 ± 1.10	34.52 ± 1.06	1.18 (0.44–1.91)	0.92	F_1,58_ = 23.55 < 0.001	< 0.001	0.54
Peak torque dorsiflexors (nm/kg)	23.41 ± 0.98	34.12 ± 0.81	10.71 (9.91–11.51)	< 0.001	24.49 ± 0.91	26.17 ± 0.90	1.68 (1.50–1.87)	0.84	F_1,58_ = 25.25 < 0.001	< 0.001	0.43
Peak torque plantar flexors (nm/kg)	38.06 ± 0.65	47.46 ± 0.90	9.40 (8.32–10.48)	< 0.001	36.62 ± 0.96	41.38 ± 0.94	1.76 (1.58–1.94)	0.84	F_1,58_ = 20.21 < 0.001	< 0.001	0.27

Values are presented as mean ± standard error of the mean (SEM).

VAS, Visual Analogue Scale; ROM/DF°, Range of motion dorsiflexion; ROM/DF°, Range of motion plantarflexion; SF-36 (PCS), Short Form 36 (SF-36) Health Survey Physical Component Summary; SF-36 (MCS), Short Form 36 (SF-36) Health Survey Mental Component Summary; MD, Mean Difference; CI, Confidence Interval.

*P*^a)^-value within a group from Paired-Samples *T* Test.

*P*^b)^- value between control and experimental group post-intervention from Independent Samples *T* Test.

*p value ≤ 0.05: statistically significant.*

*η2, partial eta square between control and experimental group post-intervention.*

Group X time interaction *p* value from two-way repeated measures ANOVA.

Table 3 shows an analysis of variation in clinical and biomechanical measures from baseline according to the severity of fracture and patient age. The results showed that generally, all clinical parameters improved toward mobilization and balance exercises compared to home-based exercise. Regarding the impact of the severity of fracture and age on the range of ankle motion post-intervention, it was noticed that patients with more severe fractures and patients ≥40 years favored mobilization and balance exercises to improve both DF and PF ROM in the experimental group. Moreover, SF-36 (MCS) was improved in patients with more severe fractures and patients <40 in the experimental group.

**Table 3:** Analysis of change in clinical and biomechanical measures from baseline according to severity of fracture and patient age.

	Mean (SEM) measure		
Variables	Experimental group	Control group	Mean difference (95% CI)
VAS (cm)			
Less severe fracture	2.43 (0.28)	6.04 (0.34)	−3.61 (−4.50– −2.72)
More severe fracture	2.88 (0.35)	5.75 (0.83)	−2.88 (−4.95– −1.13)
Age < 40 years	2.65 (0.30)	5.85 (0.47)	−3.20 (−4.29– −2.11)
Age ≥ 40 years	2.35 (0.33)	6.15 (0.38)	−3.80 (−4.89– −2.72)
ROM/ DF (degree)			
Less severe fracture	12.92 (0.45)	6.43 (0.30)	6.48 (5.40–7.56)
More severe fracture	13.31 (0.70)	5.07 (0.58)	8.25 (6.17–10.32)
Age < 40 years	12.57 (0.47)	5.96 (0.42)	6.61 (5.31–7.90)
Age ≥ 40 years	13.90 (0.56)	6.42 (0.34)	7.51 (6.21–8.81)
ROM/ PF (degree)			
Less severe fracture	24.95 (0.44)	15.21 (0.39)	9.74 (8.56–10.93)
More severe fracture	25.35 (0.92)	14.02 (0.67)	11.33 (8.69–13.80)
Age < 40 years	24.99 (0.46)	14.65 (0.44)	10.33 (9.01–11.65)
Age ≥ 40 years	25.20 (0.78)	15.38 (0.56)	9.82 (7.88–11.75)
SF-36 (PCS)			
Less severe fracture	43.38 (0.92)	33.76 (1.29)	9.62 (6.38–12.87)
More severe fracture	42.53 (1.60)	30.20 (1.61)	12.33 (7.27–17.38)
Age < 40 years	43.40 (0.98)	35.01 (1.40)	8.39 (5.00–11.78)
Age ≥ 40 years	42.66 (1.37)	30.48 (1.55)	12.18 (7.72–16.63)
SF-36 (MCS)			
Less severe fracture	44.67 (0.83)	34.73 (1.16)	9.94 (7.01–12.87)
More severe fracture	45.95 (1.18)	33.67 (2.74)	12.28 (6.38–18.19)
Age < 40 years	45.18 (0.81)	34.13 (1.52)	11.05 (7.69–14.41)
Age ≥ 40 years	44.69 (1.31)	35.02 (1.48)	9.67 (5.42–13.92)
Peak torque dorsiflexors (nm/kg)			
Less severe fracture	35.08 (0.94)	25.17 (0.91)	9.91 (7.26–12.55)
More severe fracture	31.49 (1.25)	30.17 (2.03)	1.32 (−3.62–6.26)
Age < 40 years	33.57 (0.87)	24.89 (1.20)	8.67 (5.72–11.62)
Age ≥ 40 years	35.23 (1.73)	27.84 (1.26)	7.39 (3.06–11.72)
Peak torque plantar flexors (nm/kg)			
Less severe fracture	47.61 (1.09)	41.17 (1.01)	6.44 (3.44–9.44)
More severe fracture	47.05 (1.67)	42.2 (2.56)	4.83 (−1.54–11.20)
Age < 40 years	47.81 (1.15)	41.46 (1.21)	6.35 (2.95–9.74)
Age ≥ 40 years	46.77 (1.49)	41.27 (1.54)	5.50 (0.95–10.05)

VAS, Visual Analogue Scale; ROM/DF°, Range of motion dorsiflexion; ROM/DF°, Range of motion plantarflexion; SF-36 (PCS): Short Form 36 (SF-36) Health Survey Physical Component Summary; SF-36 (MCS), Short Form 36 (SF-36) Health Survey Mental Component Summary; values are shown as mean (standard error of mean (SEM)); CI, Confidence Interval.

Patients with less severe fractures and those aged ≥40 years showed greater improvement in VAS and DF peak torque, while patients <40 years had better PF peak torque outcomes in the experimental group. Moreover, SF-36 (PCS) was improved in patients with less severe fractures and patients <40 years in the experimental group.

**Figure 3:**
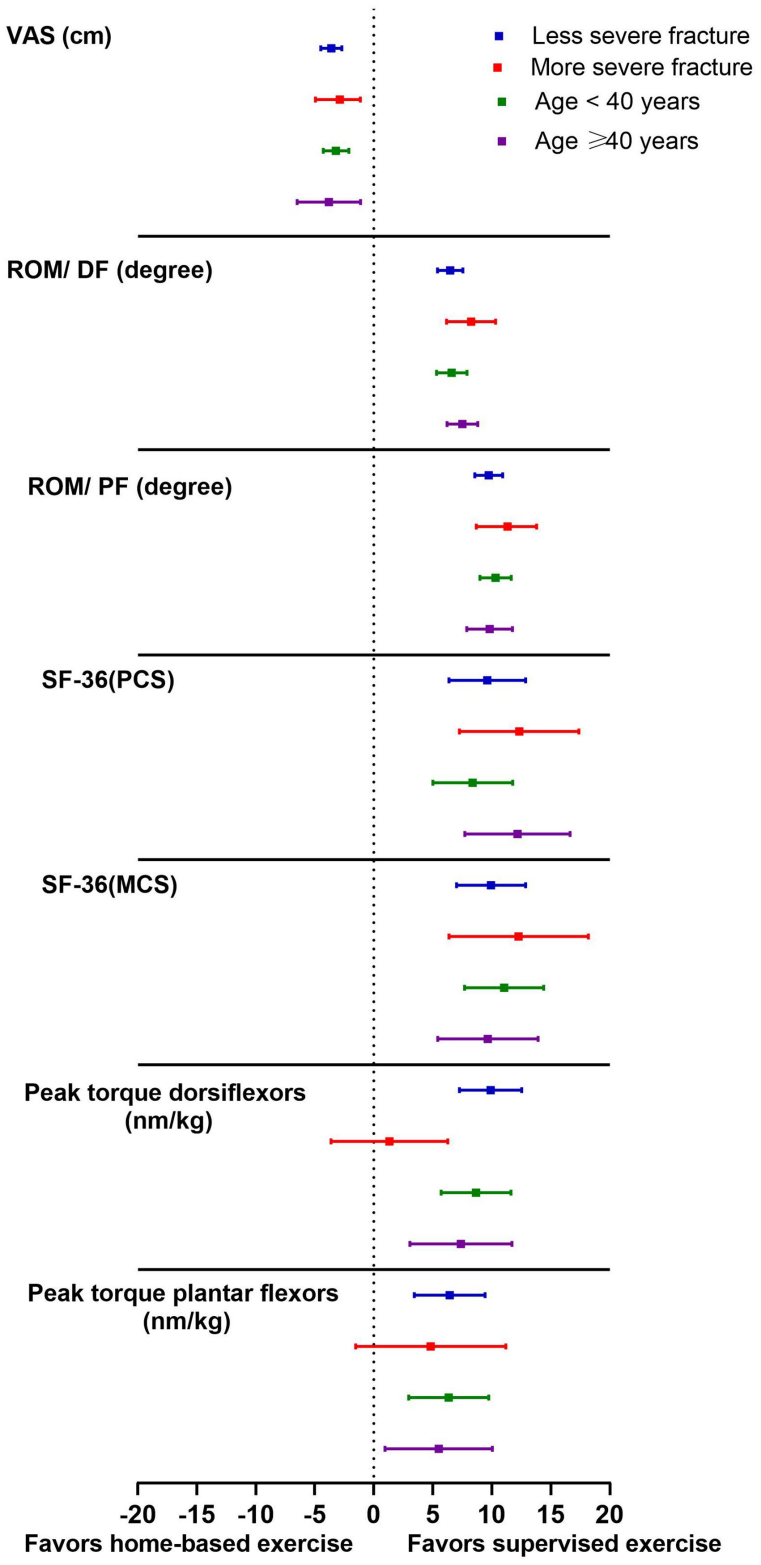
Analysis of change in clinical and biomechanical measures from baseline according to severity of fracture and patient age. Data are presented as mean differences (95% CI) between experimental and control groups.

Figure 3 shows all clinical parameters improved more in the mobilization and balance exercise group compared to the home-based exercise group, as indicated by the mean difference (95% CI) between the experimental and control groups.

## 4 Discussion

The results of the current study revealed that adding mobilization and balance to the physical therapy program was more beneficial in relieving pain, improving ankle DF and PF ROM, enhancing HRQoL, and increasing peak torque for DF and PF muscles than the home-based exercise program (*p* < 0.01).

The mechanism underlying the positive effects of mobilization and balance exercises is unclear but may involve restoring ankle motion, improving foot-ankle mechanics, reducing pain, and enhancing function. It might have a neurophysiological effect (47). It can stimulate mechanoreceptors, thus improving the neural feedback, which might aid dynamic stability and maximize exercise benefits (48). In addition, it could produce a reduction in inflammatory cytokines (49), increase beta-endorphins (50), and consequently, hypoalgesia (51). Parashkevova and Deleva (52) reported that 8 weeks of a program combining exercises with manual mobilization techniques after an ankle fracture treated with surgery had a beneficial effect in returning patients to normal work and household activities through the restoration of the arthrokinematics of the ankle-foot complex. Moreover, 3 weeks of a physical therapy program, including mobilization techniques, strength exercise, proprioceptive training, gait training, as well as Mulligan’s movement and ultrasound therapy program, showed improvement in ankle DF range, strength, as well as the overall functioning of a case report with chronic trimalleolar postoperative ankle fracture (53). General functioning and ankle dorsiflexion strength were improved after a 3-week protocol of mobilizing exercise, resistance, gait, and proprioceptive training combined with ultrasound therapy for ankle post-operative trimalleolar fracture (54).

The study’s findings on muscle strength, ROM, pain, and HRQoL align with previous research (8, 22, 33, 52, 53, 55–60). Moseley et al. (33) concluded that passive stretching exercise was more beneficial than usual in improving functional outcomes and ankle ROM after the cast removal in patients with a postoperative equinus contracture. Nilsson et al. (55) observed superior results with exercise compared to usual care concerning muscular strength and subjectively scored function after surgery post ankle fracture; this partial contradiction may be related to differences in the target population as well as the duration and protocol of treatment. Active controlled motion for the first six postoperative weeks for unstable ankle fractures hastened return to work and improved functional and clinical results (22).

Painter et al. (8) reported a positive and clinical improvement in pain, ROM, and self-reported function after stable ankle fractures post-immobilization. Improvements might be attributed to an intensive manual therapy program, the passage of time that aids the healing process, and patient-unique factors. Shaffer et al. (56) proved that 10 weeks of supervised physical therapy exercise after 2 months of cast immobilization improved function, muscle strength, and fatigue resistance. Another study concluded that the greatest muscle activation that resulted in muscle strength recovery was observed as early as the first 5 weeks of rehabilitation post-immobilization (57). Additionally, physical therapy intervention proved to be helpful for patients with chronic ankle disability (58, 59).

A well-planned post-operative trimalleolar fracture functional exercise resulted in symptom reduction, fear of motion, increased function, and improved HRQoL (60).

The study results contrasted with previous research (15, 20, 25, 27, 30, 54–57, 61–63). Two studies (27, 61) reported no additional benefits of a manual therapy program or supervised exercise program regarding activity limitation or HRQoL for ankle fractures with or without surgical fixation (61) or when comparing an exercise therapy program (2 sessions per week in the 1st week followed by one session from the 2nd to the 4th week) with an advice program (a single self-management advice session concerning exercise and return to work) for patients with isolated/uncomplicated fractured ankles (27). The contradiction might be referred to treatment duration, study protocol differences, different outcome measures (61), or differences in study design (pragmatic design) and the early termination of the advice group (single session) (27).

However, Büker et al. (62) found that pain, ankle ROM, pain disability index, QOL, and surgical satisfaction had similar clinical outcomes either with a supervised exercise program or home program in post-operative isolated fractured ankles. The contradiction might be because the study lacked randomization and the difference in the number of patients between the study and control groups. Concerning QoL, Bhandari et al. observed reduced physical function in post-operative treated fractured ankles; after 2 years of follow-up (15), patients showed. Obremsky et al. also observed decreased physical function after injury by 20 months compared to the U.S. norms in patients with an average age years after a 2-year post-surgery follow-up (63). There was no additional improvement in ROM, balance, or gait when comparing gait training combined with manual exercise with mobilization of soft tissue and the proximal tibiofibular joint after open reduction and internal fixation for fractured hindfoot or ankle. This contradiction might be referred to by the different study populations and the short-term duration of the treatment protocol (3 sessions) (25).

There are conflicting findings from earlier studies on how age affects functional outcomes. Nilsson et al. (55) revealed that rehabilitation might be more effective in older women (greater than 50 years old); however, Moseley et al. (27) demonstrated that the efficacy of rehabilitation was not influenced by age. In the present study, improvement in range of ankle motion, VAS, and dorsiflexors peak torque was observed post-intervention in the experimental group in patients ≥40 years. On the other hand, patients younger than the age of 40 years showed better improvement in peak torque plantar flexors, SF-36 (PCS), and SF-36 (MCS). Büker et al. (62) demonstrated that the scores for SF-36, including role physical, physical functioning, and role emotional, were more significant in participants under 40. Previous results reported that elderly people require longer time for functional recovery and longer time for training periods; they may need extra motivation and self-efficacy that influences functional recovery (64, 65).

### 4.1 Clinical implementation

The addition of mobilization and balance exercise to the physical therapy manual program post-RTAs for ankle joint disability had led to more contact time with health professionals; therefore, patients did the exercise precisely and more effectively, and it was likely to produce no harmful effects or adverse events. Other factors that may lead to increased exercise compliance include flexibility in session timing, constant supervision, parking, and transportation facilities. The intervention was conducted in close monitoring to guarantee both exercise safety and the dose intensity to yield the greatest benefits.

### 4.2 Limitation of the study

This study has a few limitations: Outcome measures were assessed only after 2 months of treatment. Future studies should assess the long-term effects of the programs and extend the treatment duration by more than 8 weeks to determine when participants regain normal function. In addition, other assessment methods should be included in future studies, such as electromyography (EMG), balance, and inversion and eversion ROM. The study focused solely on post-RTA ankle disability. Future research should include patients with other causes of ankle disability. In addition, the study did not explore patient-reported experiences or satisfaction with the intervention. It is recommended that future studies should implement a survey or any other method to explore patient-reported experiences or satisfaction with the intervention.

## 5 Conclusion

Adding mobilization and balance exercises to an 8-week physical therapy program for post-RTA ankle disability may significantly improve pain, ankle dorsiflexion and plantarflexion ROM, muscle torque, and quality of life compared to a standard home-based exercise program.

## Data Availability

The original contributions presented in the study are included in the article/Supplementary material, further inquiries can be directed to the corresponding author.
