# Supplementary material for: Efficacy of adding mobilization and balance exercises to a home-based exercise program in patients with ankle disability: a randomized controlled trial
Source: Front Med (Lausanne). 2025 Feb 19;12:1512587. doi: 10.3389/fmed.2025.1512587 (PMC11880611; doi:10.3389/fmed.2025.1512587)
Supplement: Supplementary file 1 [file Table_1.docx]

**Table SI. Description of physical therapy exercise program used in the study.**

| **Item** | **Description of exercise** |
| --- | --- |
| **I) Mobilization Techniques (Maitland Techniques).** | 1. **Talocrural Distraction**:   **Patient Position**:  Supine, with the lower extremity extended. Begin with the ankle in resting position. Progress to the end of the available range of dorsiflexion or plantarflexion  **Therapist Position and Hand Placement:**  ■ Working from the end of the table, place the lateral hand across the dorsum of the foot to apply a grade I distraction.  ■ Place the web space of the other hand just distal to the mortise on the posterior aspect of the talus and calcaneus.  **Mobilizing Force:**  The therapist is Pushing against the calcaneus in an anterior direction (with respect to the tibia); this glides the talus anteriorly.  **Alternate Position:**  ■ Patient is supine. The therapist stabilizes the distal leg anterior to the mortise with his proximal hand.  ■ The distal hand of the therapist cups under the calcaneus.  ■ When the therapist pulls against the calcaneus in an anterior direction, the talus glides anteriorly.   1. **Talocrural Dorsal (Posterior) Glide**   **Patient Position:**  Supine, with the affected leg supported on the table and the heel over  the edge.  **Therapist Position and Hand Placement:**   - The therapist stands to the side of the patient. stabilizes the leg with his hand or use a belt to secure the leg to the table. Placing the palmar aspect of the web space of his other hand over the talus just distal to the mortise. - Wrapping the fingers and thumb around the foot to maintain the ankle in resting position. Grade I distraction force is applied in a caudal direction.   **Mobilizing Force**  Glide the talus posteriorly with respect to the tibia by pushing against the talus.   1. **Talocrural Ventral (Anterior) Glide:**   **Patient Position:**  Prone, with the foot over the edge of the table.  **Therapist Position and Hand Placement:**   - Working from the end of the table, place his lateral hand across the dorsum of the foot to apply a grade I distraction. - Place the web space of his other hand just distal to the mortise on the posterior aspect of the talus and calcaneus.   **Mobilizing Force:**  Push against the calcaneus in an anterior direction (with respect to the tibia); this glides the talus anteriorly. |
| **II) Stretching Exercise:** | **A) Assisted calf stretching from supine:**   - **Participant Position**: Supine position. - **Therapist Position:** Stride standing beside the affected lower extremity. - **Stretching technique:** The therapist pushing the participant's knee down to lock it in a straightened position. Apply dorsiflexion force for the ankle with the other hand till the patient feels pain, maintain the force for 30 sec. Exercise can be progressed with different degrees of hip flexion.   **B) Active Stretching Exercise:**   1. **Wall stretch:**   The participants were asked to stand facing a wall, with the lower limb to be stretched extended behind and the contralateral leg placed in front. Keeping the trunk in an upright position, the participants were asked to flex the knee of their front leg until they felt a stretch in the triceps surae of the back leg, keeping the heel on the ground during the stretching, hold for 30 sec.   1. **Wall stretches on an inclined board.**   Standard board was used, with a width of 27.5 cm, length of 11 cm, and height of 7.5 cm, forming an inclination angle of 30°. The participants were asked to stand with both feet on the inclined board, with the heels placed at the lower edge of the board. They were instructed to lean forward with their hands on the wall and then to bend their elbows until they felt a stretch in the triceps surae bilaterally, hold for 30 sec.   1. **Stretching with TheraBand**:   **Participant Position:** Sit down on the floor, Loop a towel around the forefoot.  **Therapist position:** Stride standing beside the affected limb, try to keep the participant's knee completely straight by pushing the knee down.  **Technique:** The participant Pulls the towel with his hands, till feeling a stretch in the back of the calf, hold for 30 sec. |
| **III) Strengthening exercises** | **A) TheraBand exercises:**  **Patient position:**  Sitting on the floor with one end of the tubing tied around a treatment table and the other end around the metatarsal heads of the involved foot. The knees were fully extended, and the Thera-Band was stretched to 170% of its resting length, exercises were performed dorsiflexion, and plantar flexion direction.  **B) Heel raises standing on both feet:**  **Patient position:**  Standing with equal weight on both feet. Lift heels to stand on the balls of the feet. Return to the starting position. Relax and repeat.  **C) Heel raises standing with foot on a chair:**  **Patient position:**  Standing with the affected foot on the ground, the opposite foot on a chair in front of the participant. Put as much weight through the foot on the ground as possible. Lift heel to stand on the ball of the foot. Return to the starting position. Relax and repeat.  **D) Heel raises standing on 1 foot:**  **Participant position:**  Standing on the affected leg. Lift heel to stand on the ball of the foot. Return to the starting position. Relax and repeat.  **E) Heel raises standing on 1 foot on a step:**  **Patient position:**  Standing with the ball of the affected foot on a step or wooden block and lowering the heel towards the floor. Lift heel to stand on the ball of your foot, return to the starting position. Relax and repeat. |
| **IV) Weight bearing and Balance exercise:** | **1. Weight Bearing:**  Taking as much weight on the leg as possible: standing on 1 leg on hard surface(floor), then standing on 1 leg on a piece of foam, after that standing on 1 leg on a piece of foam with the closed eyes, loaded ankle dorsiflexion (30°)**,** plantar flexion (45°). rising on toes, rising on heels, and on stairs and walking at comfortable speed 30 m.  **2. Balance exercise:**   - Multidirectional rolling movement from standing on one leg (Non affected limb, then the affected limb) between parallel bars with eyes open, then eyes closed. - Participants were asked to assume a standing position on the 2-dimensional balance board while being asked to reach in multiple directions or perform upper limb movements to achieve weight shifting. - Participants were asked to assume a standing position on one leg on the 2-dimensional balance board while being asked to reach in multiple directions or perform upper limb movements to achieve weight shifting. |
