# Supplementary material for: Efficacy of adding mobilization and balance exercises to a home-based exercise program in patients with ankle disability: a randomized controlled trial
Source: Front Med (Lausanne). 2025 Feb 19;12:1512587. doi: 10.3389/fmed.2025.1512587 (PMC11880611; doi:10.3389/fmed.2025.1512587)
Supplement: Supplementary file 2 [file Table_2.docx]

**Table SII. Description of** **home- based exercise program used in the study.**

| **Item** | **Description of exercise** |
| --- | --- |
| **I) Stretching Exercise:** | **Calf muscle**:  **Soleus (Lower calf) :**  Participant's hands against the wall. Keeping the back leg (affected) straight, bending knees of both legs. Push heels down and slowly lean forward until a stretch is felt in the back (affected) of the calf. Hold for 10 – 15 seconds Repeat 2-3 times.  **Gastrocnemius (Upper calf):**  Participants hands against the wall. Keeping back (affected) leg straight Push heels down and slowly lean forward until a stretch is felt in the back of the calf Hold for 10 – 15 seconds Repeat 2-3 times. |
| **II) Strengthening exercises** | **Ankle muscles:**  **(a) Dorsiflexion:** pull the foot of the affected leg back toward the anterior of the leg, against the resistance of the TheraBand (while keeping knees straight), by moving the ankle. Then the participant was instructed to hold this position for 15 s. Return to neutral position. Repeat above steps 10 more times  **(b) Plantar flexion:** push the foot of the affected leg forward away from the body, against the resistance of the TheraBand (while keeping knees straight), by moving the ankle. Then the participant was instructed to hold this position for 15 s. Return to neutral position. Repeat above steps 10 more times. |
| **III) Active Range of Motion Exercise:** | The patient sits with the involved extremity crossed over the uninvolved one so the distal leg rests on the normal knee. The uninvolved hand moves the involved ankle into dorsiflexion, plantarflexion, inversion, and eversion, and toe flexion and extension. |
